# 

**Published:** 1850-09

**Authors:** 


Dr. S. Hanbury Smith, Professor of Medicine in the Starling Medical College, and the Editor of the Ohio Medical Journal, has received the appointment of Superintendent of the Ohio State Lunatic Asylum, at Columbus, in the place of Dr. Awl, resigned.

